# Testosterone in Relapsing–Remitting Multiple Sclerosis: A Case–Control Study

**DOI:** 10.3390/jcm15124401

**Published:** 2026-06-06

**Authors:** Iwona Rościszewska-Żukowska, Małgorzata Popiel, Adam Perenc, Julia Rudnicka-Czerwiec, Ilona Malska, Halina Bartosik-Psujek

**Affiliations:** 1 Department of Neurology, St. Jadwiga Queen Clinical Hospital, 60 Lwowska Street, 35-301 Rzeszow, Poland; 2 Department of Neurology, Collegium Medicum, University of Rzeszow, 16C Rejtana Street, 35-959 Rzeszow, Poland

**Keywords:** multiple sclerosis, free testosterone, hypogonadism

## Abstract

**Background/Objectives**: This study aimed to evaluate the prevalence of hypogonadism in men with RRMS (relapsing–remitting multiple sclerosis) who are undergoing treatment with disease-modifying therapies (DMTs) and its association with clinical and magnetic resonance imaging (MRI) parameters. **Methods**: A total of 126 male patients with RRMS, aged 18–67 years, receiving DMTs and a group of 35 age- and BMI-matched healthy individuals were enrolled. Clinical and demographic data were collected, including neurological disability (EDSS, T25FW, 9-HTP, SDMT), and MRI findings. Symptoms of androgen deficiency and depression were assessed using ADAM questionnaire and Beck Depression Inventory, while quality of life was evaluated using MSIS-29. Serum total testosterone (TT), sex hormone-binding globulin (SHBG), and free testosterone (fT) were measured in two stored morning blood samples. **Results**: A total of 118 (93.6%) MS patients (median age of 38.8 y) had normal TT levels; only 2 (1.6%) MS patients and 2 (5.7%) healthy controls had below-normal levels. Meanwhile, low fT levels were observed in 53 (43.7%) MS patients and 12 (34.3%) controls. However, the fT level was significantly lower in the MS patients under 38 years of age than in control individuals (*p* = 0.015). Only depression from all concomitant diseases was more prevalent in MS patients with low fT (*p* = 0.016). There was no correlation between low fT and clinical (EDSS, T25FT, SDMT) and MRI (new T2 and new Gd+ lesions) parameters but a longer disease duration and a higher total number of steroid treatments were associated with below-normal fT levels (*p* = 0.048 and *p* = 0.003, respectively). Change of DMT and current and previous DMT type did not correlate with low fT. Patients with low fT levels had a higher median score on the BDI (8.00; IQR: 3.00–12.50 vs. 5.00; IQR: 1.00–10.50) (*p* = 0.034) and the higher median ADAM questionnaire score (4.00 [IQR: 2.00–7.50] vs. 2.00 [IQR: 0.00–6.00]) (*p* = 0.034). Only a longer duration of MS (11.83 years) exhibited a significant positive correlation with the risk of a low fT level (OR = 1.19, CI 95%: 1.06–1.35, *p* = 0.004) in multivariate logistic analysis. **Conclusions:** Total testosterone level was normal in most male RRMS patients; however, low free testosterone level was observed, in particular in younger MS patients. Depression was more prevalent in patients with low fT but longer duration was a significant risk factor for a low fT level.

## 1. Introduction

Multiple sclerosis (MS) is a chronic inflammatory demyelinating disease of the central nervous system characterised by autoimmune-mediated damage to myelin and progressive neurodegeneration. MS is three times more prevalent in women than in men [1,2]. Significant sex differences in inflammatory activity and disease progression have also been documented. Although men experience later symptom onset and less relapse activity, they frequently exhibit accelerated disease progression, increased brain atrophy, and more pronounced cognitive impairment relative to women. Notably, gonadal hormones have been implicated in the modulation of these sex differences [3,4]. Studies have suggested that the natural, age-related decline in testosterone levels contributes to these sex-specific clinical differences in MS. Unlike the well-defined process of menopause, which is characterised by complete gonadal failure, testosterone levels in men do not decline abruptly; instead, this reduction occurs gradually over many years [5,6]. Serum testosterone exists in three forms: bound to sex hormone-binding globulin (SHBG), bound to albumin, or in a free state. The free fraction and, to some extent, the albumin fraction represent the biologically active component, capable of penetrating cells and exerting their effects through the androgen receptor [7,8].

Testosterone plays a key role in the regulation of reproductive functions, muscle mass, bone mineral density, and cognitive function. An increasing body of data suggests that testosterone may also have immunomodulatory and neuroprotective effects. This hormone influences immune system activity by attenuating the severity of inflammatory responses and supporting remyelination processes [5,9]. Studies have reported that a subset of men diagnosed with MS have reduced levels of testosterone. This phenomenon may be associated with increased symptom severity, accelerated disability progression, and a deterioration in the quality of life.

Hormonal disorders have been demonstrated to exacerbate fatigue, low mood, and sexual dysfunction, which already represent significant challenges in this patient population [10]. The objective of this study was to evaluate the prevalence of hypogonadism among males diagnosed with MS who are undergoing treatment with disease-modifying therapies (DMTs) at the MS treatment centre at KSW in Rzeszow, Poland. Potential correlations between hypogonadism and various clinical and magnetic resonance imaging (MRI) parameters were also explored.

## 2. Materials and Methods

### 2.1. Study Group

**Figure 1:**
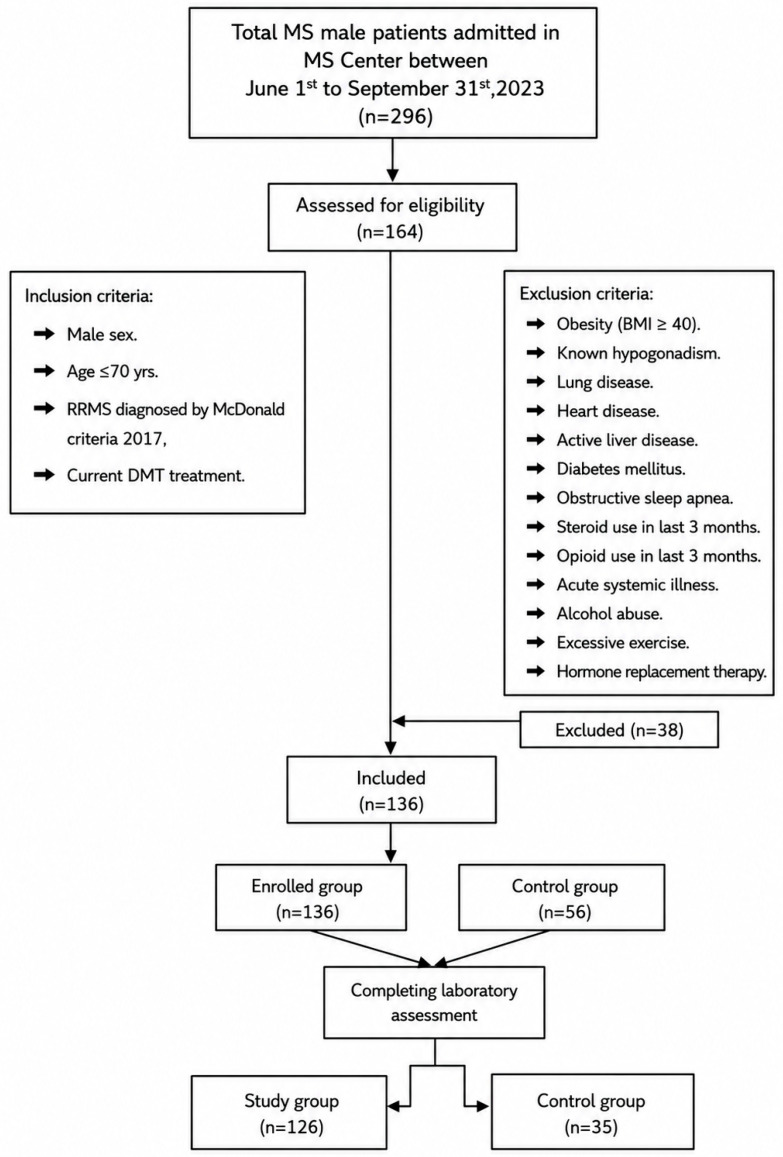
Study flow chart—study group selection.

A total of 126 male patients with relapsing–remitting multiple sclerosis (RRMS) receiving DMTs who were admitted to the MS Center of St. Jadwiga Queen Clinical Hospital in Rzeszow, Poland, between 1 June and 31 September 2023, were enrolled. A group of 35 healthy individuals served as controls. Patients with known hypogonadism, significant obesity (BMI ≥ 40), or specific concomitant diseases were excluded from the study. The exclusion criteria also included the use of opioids or steroids in the previous 3 months, as these can lower testosterone levels. Detailed inclusion and exclusion criteria are shown in Figure 1. A total of 296 male patients with MS admitted to the MS Centre were initially assessed, of whom 164 fulfilled the inclusion criteria. Of these, 38 were excluded for severe obesity (*n* = 2), heart disease (*n* = 14), lung disease (*n* = 3), liver disease (*n* = 3), sleep apnoea (*n* = 3), steroid use (*n* = 7) or opioid use (*n* = 4) in the last 3 months, or acute systemic illness (*n* = 2). Some patients (*n* = 136) and healthy controls (*n* = 56) did not provide two blood samples and were also excluded. Finally, 126 participants remained in the study group and 35 in the control group (Figure 1). The control group comprised men from the Neurology Department with no history of chronic diseases who were admitted with a diagnosis of acute lumbar pain and matched to the MS patients in a randomised fashion based on age and BMI.

The study was conducted in accordance with the Declaration of Helsinki (1964) and its subsequent amendments. The study protocol was approved by the Ethics Committee of the University of Rzeszow (protocol number 2/02/21). Written informed consent was obtained from all subjects.

### 2.2. Study Design

According to the study protocol, assessments included demographic characteristics (age, smoking status, alcohol and coffee consumption, comorbidities, and number of children) and biometric parameters (weight and BMI). Neurological status was assessed using the Expanded Disability Status Score (EDSS), timed 25-foot walk (T25FW), nine-hole peg test (9-HPT), and Symbol Digit Modalities Test (SDMT). Disease-related patient characteristics were also collected, comprising disease duration, EDSS at MS diagnosis, total number of steroid treatments, relapse frequency and symptomology, and time and type of current and past DMTs. The presence of new/enlarged T2 lesions and new gadolinium-enhancing T1 lesions in brain MRI scans performed in the last 12 months was also recorded.

Patients were screened for symptoms of testosterone deficiency using the standard self-report measure, namely, the Androgen Deficiency in Ageing Males (ADAM) questionnaire, a 10-item screening tool with high sensitivity for detecting symptoms associated with androgen deficiency. This questionnaire comprises a series of questions that assess various symptoms associated with testosterone deficiency, such as diminished libido, erectile dysfunction, decreased energy, and changes in mood. A positive ADAM result was defined as an affirmative response to either question 1 (decreased libido) or question 7 (reduced erectile strength), or to any three of the remaining questions, in accordance with established scoring criteria [11].

The Beck Depression Inventory (BDI) test was used to screen for symptoms of depression. This test comprises 21 groups of statements, which are rated from 0 to 3 points according to symptom intensity. The total score ranged from 0 to 63 points, with higher values indicating greater levels of depression. Depression was present if the score was greater than 12. The severity of depression was classified as mild (12–26 points), moderate (27–49 points), or severe (50–63 points). The Multiple Sclerosis Impact Scale (MSIS-29) was used to assess impact of MS on daily live; it is a 29-item self-report questionnaire assessing the physical (20 items) and psychological (9 items).

### 2.3. Hormonal Measures

Blood samples were collected to determine the levels of total testosterone (TT), sex hormone-binding globulin (SHBG), and free testosterone (fT) in the blood. The samples were collected on two separate mornings between 07:00 and 10:00 h. The fT levels were calculated using the serum values for TT, SHBG, and albumin using the Vermeulen formula. In patients with low TT or normal TT and low fT, luteinizing hormone (LH) and follicle-stimulating hormone (FSH) levels were measured to exclude primary hypogonadism.

### 2.4. Statistical Analysis

To estimate the association between the studied parameters and the occurrence of hypotestosteronemia, univariate analysis was performed. Subsequently, to identify risk factors for hypotestosteronemia in a group of MS patients, a multivariate analysis was employed.

## 3. Results

### 3.1. Patient Characteristics

A total of 126 men with RRMS and 35 controls without MS were assessed in the study. The mean age of the study group was 38.8 ± 10.9 years (range: 18–67), while that of the control group was 45.3 ± 7.9 years (range: 20–69) (*p* = 0.004). The median age was the same in both groups (41.0 years). There was no statistically significant difference between the BMI of the test and control groups. More than 30% of MS patients had concomitant diseases, with hypertension and depression being the most common. Additionally, 39.3% of patients from the MS group and 45.7% of the controls were childless (*p* = 0.357). At the time of the study, the mean EDSS of patients with MS was 2.2 (SD = 1.1), and the mean time since diagnosis was 4.7 years (SD = 3.6). In the MS group, the most frequently used DMTs were interferons and dimethyl fumarate. The detailed characteristics of the study and control groups are shown in Table 1.

### 3.2. Hormonal Assessment

**Figure 2:**
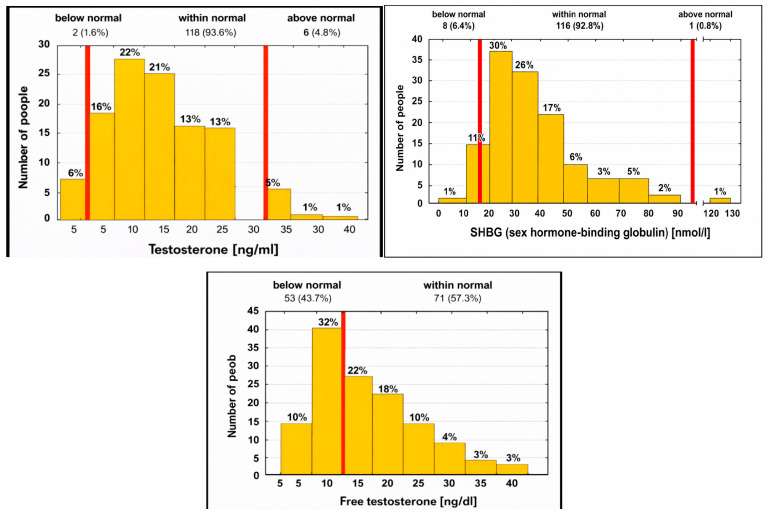
Percentage and number of individuals with MS whose total testosterone (TT), sex hormone-binding globulin (SGBG), and free testosterone (fT) levels were within, below, or above the reference range.

A total of 118 patients (93.6%) had normal TT levels, 2 (1.6%) had below-normal levels, and 6 (4.8%) displayed above-normal levels. SHBG levels were normal in 116 (92.8%) of the MS patients, low in 8 patients (6.4%), and high in 1 patient (0.8%). Free testosterone levels were low in 53 patients (43.7%) and normal in 77 patients (57.3%). No patient had an above-normal fT level (Figure 2). There were no statistically significant differences in hormone levels between the study group and the control group. The median TT, SHBG, and calculated fT levels are presented in Table 2. Two MS patients (1.6%) and two controls (5.7%) had low TT levels (*p* = 0.352). While low fT levels were more frequently observed in MS patients than in controls, this difference did not reach statistical significance (53 patients [43.7%] vs. 12 controls [34.3%]), *p* = 0.368).

### 3.3. Clinical Factors and Hormonal Status

The median age did not differ significantly between the low-fT and normal-fT groups, with both cohorts averaging 41 years (IQR: 32.50–47.50 vs. IQR: 30.25–47.00, *p* = 0.352). Additionally, no correlation was found between the age of MS patients and their TT, fT, or SHBG levels. However, the fT level was significantly lower in the subgroup of MS patients under 38 years of age than in control individuals in the same age subgroup (*p* = 0.015) (Table 3).

The median BMI was 25.62 (IQR: 23.57–28.94) in the low-fT group, and 24.29 (IQR: 22.76–27.69) in the normal-fT group (*p* = 0.127).

The prevalence of obesity, hypertension, hypothyroidism, hyperlipidaemia, and asthma was comparable between the low-fT and normal-fT groups. However, depression was more prevalent in patients with low fT (*p* = 0.016). Patients with low fT levels did not consume alcohol or coffee. However, smoking was more prevalent in this group than in patients with normal fT levels (*p* = 0.084) (Table 4).

No correlation was observed between clinical MS parameters—such as EDSS at diagnosis, current EDSS, T25FW, and SDMT—and low levels of fT. Similarly, low fT levels were not correlated with the findings of new T2 lesions or new Gd+ lesions on MRI scans. Conversely, a longer disease duration and a higher total number of steroid treatments were found to be associated with below-normal fT levels (*p* = 0.048 and *p* = 0.003, respectively). The impact of MS on quality of life, as measured by the MSIS, did not differ between the low-fT and normal-fT groups. Patients with low fT levels had a higher median score on the BDI (8.00; IQR: 3.00–12.50) than those with normal fT levels (5.00; IQR: 1.00–10.50) (*p* = 0.034). In the low-fT group, 30.91% of MS patients had a BDI above 11—suggestive of clinically significant depressive symptoms—compared to 22.54% in the normal-fT group. However, this difference was not statistically significant. The median ADAM questionnaire score was higher in the low-fT group than in the normal-fT group (4.00 [IQR: 2.00–7.50] vs. 2.00 [IQR: 0.00–6.00]) (*p* = 0.034). Nevertheless, the percentage of patients with more than three positive ADAM items did not differ between the two groups (Table 4).

### 3.4. DMTs and Testosterone

The median duration of current DMT therapy for the whole cohort was 5.96 years; however, no difference in this parameter was observed between the low-fT and normal-fT groups. Moreover, a greater number of patients were treated with natalizumab in the low-fT group than in the normal-fT group (14.5% vs. 4.2%, *p* = 0.057), and a similar result was obtained for interferon treatment (16.4% vs. 29.6%, *p* = 0.084); however, these findings did not reach statistical significance. For other therapies, no significant differences were found between patients with and those without low fT levels. Although in a few cases numerical differences were apparent—for instance, regarding INF beta 1 a i.m., INF beta 1 a s.c., and cladribine—these differences did not reach statistical significance, potentially due to the small sizes of the individual treatment groups.

A change of treatment was recorded in 40.5% of all MS patients. In the group with low fT levels, 49.1% of patients changed their therapeutic regimen; in the group with normal fT levels, 33.8% of patients changed their treatments. The difference between the groups approached, but did not attain, statistical significance (*p* = 0.083) (Table 5).

### 3.5. Predictors of Low fT Levels in MS Patients

To identify risk factors for low fT levels in MS patients, a logistic regression model was constructed using several selected variables known to influence this risk. This model, characterised by a R^2^*_Tjur_* value of 0.606, demonstrated a high capacity to explain the variability in low fT prevalence in the MS patient cohort. The results of the model are shown in Table 6.

The findings indicated that the study group exhibited a significant baseline risk of low fT levels, which was calculated at 23.7%. Total testosterone levels, centred on a median of 4.31 ng/mL, showed a strong inverse relationship with the occurrence of low fT levels.

The findings of this study indicated that decreased TT levels were significantly associated with an elevated risk of low fT levels (OR = 0.10, 95% CI: 0.03–0.22, *p* < 0.001). High levels of SHBG, centred on a median of 33.14 nmol/L, were also found to be significantly associated with a higher risk of low fT levels (OR = 1.13, CI 95%: 1.07–1.22, *p* < 0.001). Similarly, a longer duration since MS diagnosis, centred on a median of 11.83 years, exhibited a significant positive correlation with the risk of a low fT level (OR = 1.19, CI 95%: 1.06–1.35, *p* = 0.004).

While obesity was associated with a reduced risk of low fT levels, the result did not reach statistical significance. Furthermore, we found an association between depression and a significantly elevated risk of low fT levels. However, this result was also on the borderline of statistical significance.

## 4. Discussion

The literature regarding hypogonadism in men with MS remains inconsistent [12]. While some studies have reported a high prevalence, affecting over 40% of men aged 18 to 40 [10], others have found no significant differences compared to the general population [13,14]. In the present study, TT levels were within the normal range for most patients with RRMS, whereas low fT levels were observed in approximately 43% of patients. These data align with findings by D’Amico et al., 2020 who reported that patients with RRMS did not show abnormalities in gonadal steroid profiles, except for fT levels in the study group relative to the control group [14].

In our study, we found no significant differences in TT, SHBG, or fT levels between the test and control groups. However, the observation that a relatively high proportion of patients exhibited lowered fT alongside normal TT levels highlights the importance of determining the biologically active fraction of testosterone in the men studied. Because fT has a direct effect on the androgen receptor, a decrease in its levels may have clinical significance despite normal TT values. In the young male MS cohort, almost half of the MS patients met the criteria for a positive result on the ADAM questionnaire. However, no differences were found between the group with low free testosterone levels and the group with normal levels. Consequently, it appears rational to evaluate serum testosterone levels in young men with MS, even in the absence of symptoms indicative of hypogonadism.

Furthermore, research by Bove et al. suggested that an association exists between low testosterone levels and increased disability and cognitive impairment [10]. We did not identify an association between low fT levels and the current degree of neurological disability (EDSS), gait performance (T25FW), cognitive function (SDMT), or inflammatory activity on MRI scans. These data suggest that reduced fT does not directly indicate current disease activity or the extent of neurological damage. Similar results were reported by Diaconu et al., 2022 [15]. Aside from the expected inverse relationship with BMI, testosterone levels did not correlate with demographic or disease-specific factors.

The duration of either the current treatment or previous DMTs did not appear to be associated with reduced fT levels. While natalizumab was more frequently administered in the group with reduced fT and interferon beta-1b levels than in the group with normal fT levels, these results require cautious interpretation due to the small size of the study group. Such findings may reflect the fact that natalizumab was used for the treatment of patients with a more severe disease course. Additionally, the more frequent change of therapy in the low-fT group suggests that a link exists between androgen disruption and a more dynamic or difficult-to-control disease progression. The potential association between the type of therapy and androgen metabolism requires confirmation in larger studies.

A significant factor associated with low fT levels was a longer duration of MS. Each additional year of disease increased the risk of a lower fT fraction. This result may reflect the chronic impact of the inflammatory process and the cumulative effect of biological stress, as well as the potential disruption of the hypothalamic–pituitary–gonadal axis during long-term autoimmune disease. Pro-inflammatory cytokines such as IL-1β, IL-6, and TNF-α may impair the gonadotropin-releasing hormone (GnRH) secretion in the hypothalamus, reduce luteinising hormone (LH) secretion by the pituitary gland. GnRH and directly affect Leydig cell function, leading to reduced testosterone bioavailability [16]. Long-term activation of the hypothalamic–pituitary–adrenal axis, associated with chronic biological and psychological stress in autoimmune disease, may contribute to suppression of gonadal function [5]. Elevated cortisol levels can inhibit testosterone secretion and promote hormonal imbalance, effects that may be further exacerbated by repeated steroid therapies used to treat MS relapses [17].

Additionally, a higher number of steroid therapies was associated with lower fT levels, which is consistent with the fact that glucocorticoids suppress the endocrine axis and can disrupt androgen metabolism, and so this mechanism may explain why higher steroid treatments are linked to lower fT levels. The number of relapses between the diagnosis and last assessment could indicate inflammatory activity affecting the hypothalamic–pitutitary–gonadal axis. This parameter was left out of the analysis due to incomplete data on patients’ courses, representing a study limitation. Other factors associated with chronic neurological disease may also impact the hormonal axis. Limited physical activity, chronic fatigue, sleep disorders and vitamin D deficiency are common in patients with MS and may affect androgen metabolism and depressive symptoms.

Vitamin D deficiency is associated with the incidence of depression and chronic fatigue in MS; in middle-aged and elderly MS men positive associations between 25(OH)D and testosterone was reported and it was proposed that vitamin D could affect mood via androgen pathways and may also play a protective role in depression through its effect on the testosterone [18]. Frequent insomnia, restless legs, sleep-disordered breathing and daytime sleepiness are common in people with MS and can affect quality of life. Some studies suggest that hypothalamic–pituitary–gonadal axis abnormalities and androgen–oestrogen interplay may impact MS, possibly affecting sleep regulation and neuroprotection, though direct sleep data are lacking [12,19].

There is an increasing focus on the role of chronic psychological stress and the fear of relapse (FoR). Anxiety is often linked to concerns about the course of the disease, future disability and fatigue. Chronic emotional stress can lead to sustained activation of the stress axis and secondary hormonal disturbances. FoR is associated with more severe depressive symptoms and a reduced quality of life in patients with MS, which may also indirectly affect the functioning of the endocrine system [20].

In our study, a logistic regression model demonstrated a strong independent association between SHBG levels and the risk of low fT. An increase in SHBG concentrations significantly increased the likelihood of a lower fT fraction, which is consistent with a physiological mechanism involving greater testosterone binding and reduced bioavailability. Conversely, lower TT values, even within the reference range, were associated with a significantly higher risk of low fT, which aligns with data reported in the literature [21].

Patients with low fT levels scored higher on the BDI and were more likely to be diagnosed with depression. Although some results were borderline statistically significant, these data nonetheless suggest that an association exists between reduced testosterone levels and the severity of depressive symptoms. The relationship between depression and testosterone concentrations may be multifactorial and bidirectional [10]. Androgen deficiency may affect serotonin and dopamine neurotransmission, leading to a lowered mood. Conversely, chronic depression and psychological stress may disrupt the regulation of the hormonal axis. While our study cannot determine the direction of the cause-and-effect relationship, the results indicate the need for a more comprehensive neuropsychiatric and endocrinological assessment of men with MS.

The main limitations of our study include the relatively small size of certain treatment subgroups, which may have influenced the statistical significance of various analyses and resulted in wide confidence intervals within the regression model. Furthermore, the absence of a dynamic assessment regarding hormonal changes over time restricted our ability to evaluate the progression of androgen disorders.

The observation that men with MS have normal TT levels alongside reduced fT levels may be clinically significant. This phenomenon highlights the complex nature of androgen regulation and suggests that assessing TT alone may result in an underestimation of the prevalence of endocrine disruption. Our results suggest that, for men with long-standing MS and concomitant depressive symptoms, determining fT levels may be warranted, even when TT levels remain within the reference range.

Further studies involving larger populations and prospective follow-up of patients are needed to assess the potential impact of androgen disruption on MS progression and treatment response; interventional prospective studies should assess whether the identification of patients with biochemical testosterone deficiency, followed by an appropriate intervention—including possible testosterone replacement therapy. It is particularly important to determine whether correcting fT deficiency affects the clinical, psychological, and functional parameters of this patient group. However, the efficacy and safety of such interventions in patients with MS require further investigation.

## 5. Conclusions

Low free testosterone serum level has been observed in younger males with MS; however, total testosterone level was normal in most male RRMS patients. Lower free testosterone level can correlate with depression and longer disease duration. This suggests that measuring total testosterone alone may not identify androgenic disorders in men with MS. Measuring free testosterone could therefore be important, particularly in patients with a chronic disease course and neuropsychiatric symptoms. Further studies involving a larger patient group are needed to determine the impact of androgenic disorders on disease activity, cognitive function, quality of life, and response to testosterone replacement therapy. It is particularly important to determine whether correcting fT deficiency affects the clinical, psychological, and functional parameters of this patient group.

## Figures and Tables

**Table 1:** Characteristic of the study cohort.

Characteristics	Study Group (n = 126)		Control Group (n = 35)		*p*
	Mean (SD)	Range	Mean (SD)	Range	
Age (yrs)	38.8 (10.9)	18–67	45.3 (7.9)	20–69	0.004
Proportion of patients under 38 years of age, n (%)	60 (47.6)		13 (37.2)		0.271
	Mean (SD)	Range	Mean (SD)	Range	
BMI	25.6 (5.4)	16–38	24.1	17–39	0.438
Smoking, n (%)	70 (55.6)		20 (57.1)		
Coffee everyday use, n (%)	95 (75.4)		27 (77.1)		
Alcohol use, n (%)	51 (40.5)		14 (40.0)		
Having children, n (%)	76 (60.3)		19 (54.3)		0.357
	Present	Absent	NA		
Comorbidities, n (%)	40 (31.7)	86 (68.3)			
Hypertension, n (%)	19 (15.1)	-			
Depression, n (%)	17 (13.5)	-			
Obesity, n (%)	7 (5.6)	-			
Hypothyroidism, n (%)	4 (3.2)	-			
Hyperlipidaemia, n (%)	3 (2.4)	-			
Asthma, n (%)	2 (1.6)	-			
	Mean (SD)	Range	Mean (SD)	Range	
Disease duration from diagnosis (yrs)	4.7 (2.6)	2–14	NA		
Number of methyloprednisolon pulses, n	3.0 (2.3)	(1–16)	NA		
Time DMTs (yrs)	5.9 (2.4)	1.7–16.6	NA		
EDSS at onset of MS (points)	1.7 (1.0)	0–4.5	NA		
Actual EDSS (points)	2.2 (1.1)	1–6	NA		
EDSS change (points)	0.5 (0.9)	1–4	NA		
T25FW (s)	5.1 (2.1)	4–18	NA		
SDMT score	48.2 (13.9)	18–78	NA		
MSIS-29 score	55.3 (22.7)	29–105	NA		
BDS score	7.9 (7.7)	0–38	NA		
ADAM score	3.7 (3.1)	0–10	NA		
DMTs, n (%)					
Interferons	39.0 (31.0)	-			
Dimethyl fumarate	39.0 (31.0)	-			
Glatiramer acetate	8.0 (6.3)	-			
Teriflunomide	8.0 (6.3)	-			
Fingolimod	9.0 (7.1)	-			
Ofatumumab	8.0 (6.3)	-			
Natalizumab	11.0 (8.7)	-			
Cladribine	4.0 (3.2)	-			

SD, standard deviation; DMTs, disease-modifying treatments; EDSS, Expanded Disability Status Score; T25FW, timed 25-foot walk test; 9-HPT, nine-hole peg test, SDMT, Symbol Digit Modalities Test; MSIS-29, Multiple Sclerosis Impact Scale; BDS, Beck Depression Inventory test; ADAM, Androgen Deficiency in Ageing Males questionnaire score.

**Table 2:** The median levels of total testosterone (TT), sex hormone-binding globulin (SHBG), and calculated free testosterone (fT).

	Study Group (n = 126)		Control Group (n = 35)		
	Mean (95% CI)	SD	Mean (95% CI)	SD	
Total testosterone (TT) (nq/mL)	4.46 (4.15–4.77)	1.74	4.30 (3.64–4.95)	1.91	0.7302
Sex hormone-binding globulin (SHBG) (nmol/L)	36.30 (33.13–39.48)	17.94	34.13 (28.87–39.38)	15.29	0.7705
Free testosterone (fT) (pg/mL)	18.11 (16.83–19.38)	7.18	18.35 (15.51–21.18)	8.25	0.8409

SD, standard deviation.

**Table 3:** Impact of age on total testosterone (TT), sex hormone-binding globulin (SHBG), and calculated free testosterone (fT).

Group	Total Testosterone (TT) Level					
	<38 Age (*p* = 0.2694)			≥38 Age (*p* = 0.1753)		
	*N*	Mean (95% CI)	SD	*N*	Mean (95% CI)	SD
Study group	56	4.33 (3.85–4.82)	1.80	70	4.56 (4.15–4.96)	1.70
Control group	13	4.91 (4.03–5.78)	1.45	22	3.94 (3.01–4.86)	2.08
Group	Sex hormone-binding globulin (SHBG) level					
	<38 age (*p* = 0.6327)			≥38 age (*p* = 0.4362)		
	*N*	Mean (95% CI)	SD	*N*	Mean (95% CI)	SD
Study group	56	32.88 (28.72–37.05)	15.54		39.08 (34.43–43.73)	19.35
Control group	13	32.64 (26.69–38.58)	9.84		35.01 (27.06–42.96)	17.92
Group	Free testosterone (fT) level					
	<38 age (*p* = 0.0151)			≥38 age (*p* = 0.0958)		
	*N*	Mean (95% CI)	SD		Mean (95% CI)	SD
Study group	56	18.38 (16.26–20.51)	7.94	68	17.88 (16.29–19.46)	6.54
Control group	13	23.75 (19.43–28.07)	7.15	22	15.15 (11.95–18.36)	7.23

SD, standard deviation.

**Table 4:** Clinical factors and low free testosterone level.

Characteristics	N	Total		Low fT (n = 55)		Normal fT (n = 71)		*p*
Obesity, n (%)	126	7 (5.6)		4 (7.3)		3 (4.2)		0.698 ^b^
Hypertension, n (%)	126	19 (15.0)		12 (21.8)		7 (9.8)		0.063 ^a^
Hypothyroidism, n (%)	126	4 (3.2)		2 (3.6)		2 (2.8)		1.000 ^b^
Hyperlipideamia, n (%)	126	3 (2.4)		1 (1.8)		2 (2.8)		1.000 ^b^
Depression, n (%)	126	17 (13.5)		12 (21.8)		5 (7.0)		0.016 ^a^
Asthma, n (%)	126	2 (1.6)		0 (0)		2 (2.8)		0.504 ^b^
Smoking, n	116							0.084 ^a^
Current, n (%)		24 (20.7)		9 (18.0)		15 (22.7)		
Past, n (%)		36 (31.0)		21 (42.0)		15 (22.7)		
Never, n (%)		56 (48.3)		20 (40.0)		36 (54.5)		
Alcohol, n (%)	107	51 (47.7)		19 (40.4)		32 (53.3)		0.185 ^a^
Coffee, n (%)	120	95 (79.2)		44 (83.0)		51 (76.1)		0.355 ^a^
Family MS, n (%)	124	13 (10.5)		6 (11.1)		7 (10.0)		0.841 ^a^
BDS (>11 scores), n (%)	56	33.00 (26.2)		17.00 (30.9)		16.00 (22.5)		0.289 ^a^
ADAM (>3 scores), n (%)	126	62.00 (49.2)		31.00 (56.36)		31.00 (43.66)		0.157 ^a^
New MRI T2 lesions, n (%)	108	6.00 (5.6)		1.00 (2.2)		5.00 (7.9)		0.397 ^a^
MRI Gd+ lesions, n (%)	108	12.00 (11.1)		2.00 (4.4)		10.00 (15.9)		0.071 ^a^
		Mean (SD)	Range	Mean (SD)	Range	Mean (SD)	Range	*p*
Duration of MS (yrs)	126	11.83	7.8–17.7	13.8	9.6–18.4	10.5	7.4–16.4	0.048 ^c^
EDSS at MS onset	126	1.50	1.0–2.0	1.50	1.0–2.0	1.50	1.0–2.0	0.689 ^c^
Actual EDSS	126	2.19	1.0–3.0	2.32	1.0–3.5	2.08	1.0–3.5	0.253 ^c^
T25FW (s)	126	4.0	4.0–5.0	5.0	4.0–5.5	4.0	4.0–5.5	0.371 ^c^
SDMT score	126	51.5	37.0–59.0	44.0	35.0–55.0	55.0	37.0–60.0	0.216 ^c^
MSIS-29 score	126	52.0	35.0–69.0	55.0	38.0–76.0	48.0	33.0–66.0	0.272 ^c^
Number of relapses before diagnosis (n)	124	2.0	1.0–2.0	2.0	1.0–2.0	2.0	1.0–2.0	0.598 ^c^
Total steroid treatment numbers (n)	124	2.0 (2.3)	2.0–4.0	3.0(2.1)	2.0–4.0	2.0(2.5)	1.0–3.5	0.003 ^c^
BDS score	126	6.0	1.25–12.0	8.0	3.0–12.5	5.0	1.0–10.5	0.034 ^c^
ADAM score	126	3.0	1.0–6.0	4.0	2.0–7.5	2.0	0–6.0	0.034 ^c^

^a^ Chi-square independence test, ^b^ Fisher’s test, ^c^ Wilcoxon’s test; SD, standard deviation; BDS, Beck Depression Inventory test; ADAM, Androgen Deficiency in Ageing Males questionnaire score; EDSS, Expanded Disability Status Score; T25FW, timed 25-foot walk test; SDMT, Symbol Digit Modalities Test; MSIS-29, Multiple Sclerosis Impact Scale; ADAM, Androgen Deficiency in Ageing Males questionnaire score.

**Table 5:** Free testosterone (fT) levels in the study group categorised by DMT type.

DMTs	N	Total	Free Testosterone (fT)		*p*
			Low fT n = 55	Normal fT n = 71	
**Current Treatment**					
Time of current DMT, yrs, (range)	126	5.96 (3.19, 6.75)	6.00 (3.08, 6.75)	5.92 (3.21, 6.75)	0.688
INF B1B, n (%)	126	30 (23.8)	9 (16.4)	21 (29.6)	0.084
Peg INF, n (%)	126	3 (2.4)	2 (3.6)	1 (1.4)	0.580
INF beta 1 a i.m., n (%)	126	4 (3.2)	1 (1.8)	3 (4.2)	0.631
INF beta 1 a s.c., n (%)	126	2 (1.6)	0 (0)	2 (2.8)	0.504
Dimethyl fumarate, n (%)	126	39 (31)	18 (32.7)	21 (29.6)	0.704
Teriflunomide, n (%)	126	8 (6.3)	3 (5.4)	5 (7.0)	1.000
Ofatumumab, n (%)	126	8 (6.3)	5 (9.1)	3 (4.2)	0.295
Glatiramer acetate, n (%)	126	8 (6.3)	4 (7.3)	4 (5.6)	0.728
Fingolimod, n (%)	126	9 (7.1)	4 (7.3)	5 (7.0)	1.000
Natalizumab, n (%)	126	11 (8.0)	8 (14.5)	3 (4.2)	0.057
Cladribine, n (%)	126	4 (3.2)	1 (1.8)	3 (4.2)	0.631
Change of DMT, n (%)	126	51 (40.5)	27 (49.1)	24 (33.8)	0.083
**Previous Treatment**					
Time of previous DMT, yrs, (range)	51	6.79 (6.62, 6.81)	6.80 (6.77, 6.82)	6.77 (5.48, 6.81)	0.055
Previous DMTs	51				0.751
INF B1B, n (%)		11 (21.6)	7 (25.9)	4 (16.7)	
Peg INF, n (%)		1 (1.9)	1 (3.7)	0 (0)	
INF beta 1 a i.m., n (%)		4 (7.8)	3 (11.1)	1 (4.1)	
INF beta 1 a s.c., n (%)		16 (31.4)	8 (29.6)	8 (33.3)	
Dimethyl fumarate, n (%)		12 (23.5)	5 (18.5)	7 (29.2)	
Teriflunomide, n (%)		1 (1.9)	1 (3.7)	0 (0)	
Ofatumumab, n (%)		0 (0)	0 (0)	0 (0)	
Glatiramer acetate, n (%)		4 (7.8)	1 (3.7)	3 (12.5)	
Fingolimod, n (%)		2 (3.9)	1 (3.7)	1 (4.2)	
Natalizumab, n (%)		0 (0)	0 (0)	0 (0)	
Cladribine, n (%)		0 (0)	0 (0)	0 (0)	

Chi-square independence test, Fisher’s test, Wilcoxon’s test.

**Table 6:** Multivariate logistic regression model using the stepwise method for selection of independent predictors of low free testosterone level in MS patients, R^2^*_Tjur_* = 0.606, *N* = 112 *.

Predictive Variables	Low Free Testosterone Group		
	Odds Ratio (OR)	95% Confidence Interval	*p*
(Constant)	0.31	0.09–0.87	0.035
Testosterone Centred for *Mdn* = 4.31ng/mL	0.10	0.03–0.22	<0.001
SHBG Centred for *Mdn* = 33.14nmol/L	1.13	1.07–1.22	<0.001
Time from diagnosis Centred for *Mdn* = 11.83 lat	1.19	1.06–1.35	0.004
	**Prevalence of obesity**		
No	Reference category		
Yes	0.07	0.00–1.15	0.061
	**Prevalence of depression**		
No	Reference category		
Yes	6.30	1.03–52.27	0.063

* After taking into account only records without missing values in the range of variables analysed.

## Data Availability

The original contributions presented in this study are included in the article. Further inquiries can be directed to the corresponding author.

## Abbreviations

The following abbreviations are used in this manuscript:

ADAM	Androgen Deficiency in Ageing questionnaire
BDI	Beck Depression Inventory
DMTs	Disease-modifying therapies
EDSS	Expanded Disability Status Score
FSH	Follicle-stimulating hormone
fT	Serum free testosterone
9-HTP	Nine-hole peg test
INF	Interferon
LH	Luteinizing hormone
MRI	Magnetic resonance imaging
MSIS-29	Multiple Sclerosis Impact Scale-29
RRMS	Relapsing–Remitting Multiple Sclerosis
SDMT	Symbol Digit Modalities Test
T25FW	Timed 25-foot walk
TT	Serum total testosterone
